# Recruitment and retention of participant and study partner dyads in two multinational Alzheimer’s disease registration trials

**DOI:** 10.1186/s13195-020-00762-8

**Published:** 2021-01-08

**Authors:** Olivia M. Bernstein, Joshua D. Grill, Daniel L. Gillen

**Affiliations:** 1 Department of Statistics, University of California, Irvine, Bren Hall 2019, Irvine, CA 92697-1250 USA; 2 Institute for Memory Impairments and Neurological Disorders, University of California, Irvine, Irvine, CA USA; 3 Alzheimer’s Disease Research Center, University of California, Irvine, Irvine, CA USA; 4 Department of Psychiatry and Human Behavior, University of California, Irvine, Irvine, CA USA; 5 Department of Neurobiology and Behavior, University of California, Irvine, Irvine, CA USA

**Keywords:** Alzheimer’s disease, Clinical trials, Recruitment, Retention, Study partner

## Abstract

**Background:**

Early study exit is detrimental to statistical power and increases the risk for bias in Alzheimer’s disease clinical trials. Previous analyses in early phase academic trials demonstrated associations between rates of trial incompletion and participants’ study partner type, with participants enrolling with non-spouse study partners being at greater risk.

**Methods:**

We conducted secondary analyses of two multinational phase III trials of semagacestat, an oral gamma secretase inhibitor, for mild-to-moderate AD dementia.

Cox’s proportional hazards regression model was used to estimate the relationship between study partner type and the risk of early exit from the trial after adjustment for a priori identified potential confounding factors. Additionally, we used a random forest model to identify top predictors of dropout.

**Results:**

Among participants with spousal, adult child, and other study partners, respectively, 35%, 38%, and 36% dropped out or died prior to protocol-defined study completion, respectively. In unadjusted models, the risk of trial incompletion differed by study partner type (unadjusted *p* value = 0.027 for test of differences by partner type), but in models adjusting for potential confounding factors, the differences were not statistically significant (*p* value = 0.928). In exploratory modeling, participant age was identified as the primary characteristic to explain the relationship between study partner type and the risk of failing to complete the trial. Participant age was also the strongest predictor of trial incompletion in the random forest model.

**Conclusions:**

After adjustment for age, no differences in the risk of incompletion were observed when comparing participants with different study partner types in these trials. Differences between our findings and the findings of previous studies may be explained by differences in trial phase, size, geographic regions, or the composition of academic and non-academic sites.

## Background

Clinical trials are essential for evaluating the safety and efficacy of potential disease-modifying drugs for Alzheimer’s disease (AD) but face notable challenges [1, 2]. In particular, inefficient recruitment and challenging retention consistently delay AD trials and threaten their integrity. Barriers to recruitment include low awareness of trials, primary care physicians’ reluctance to refer patients, participants’ hesitancy to take investigational therapies and undergo invasive procedures, and strict inclusion criteria that may preclude participation for a large proportion of AD patients [3–5]. Resultantly, AD trial participants tend to be disproportionately white and with more years of education, compared to all AD patients in the USA [4–6].

All AD clinical trials require participants to enroll with a study partner. The study partner is often the participant’s primary caregiver [5]. Study partners are integral to AD clinical trial conduct—they may assist with informed consent, ensure protocol compliance, and serve as informants for cognitive, functional, and behavioral outcome measures. Most primary caregivers for people with dementia are non-spouses, in particular adult children of the person with dementia [7–9]. Adult children are more likely than spouses to be working, caring for families, and have other responsibilities besides caregiving. Thus, it may be more difficult for adult children to fulfill trial obligations in long-term studies [5].

We previously observed an association between study partner type and trial recruitment and retention in a meta-dataset composed of six trials funded by the National Institute of Aging (NIA) that enrolled participants with possible or probable AD. In these studies, 67% of patients enrolled with a spouse, 26% enrolled with an adult child study partner, and 7% enrolled with a study partner who was neither a spouse nor an adult child (herein “other”). Trial incompletion was higher among participants enrolling with a non-spouse study partner [10].

Early study exit due to dropout or death in clinical trials causes missing data. In the best case scenario, this results in a loss of statistical precision for estimated treatment effects and reduced power. In the worst case scenario, missing data can produce bias in the estimated treatment effect. Expert guidance on statistical handling of missing data is clear: the first priority should be to prevent its occurrence [11]. Understanding predictors of early study exit will allow trial investigators to better design trials and to identify and support participants at increased risk for incompletion in order to prevent missing data.

Whether the relationship between AD study partner type and trial completion is homogenous across varying trial types and designs is unknown. The phases of the drug development process have unique objectives and therefore unique study designs [12]. Additionally, site networks conducting industry-funded trials differ from academic trials and may enroll different patient populations. For example, NIA-funded mild cognitive impairment (MCI) trials may enroll larger proportions of apolipoprotein E ε4 (*APOE*4) carriers than do industry-funded trials [13]. An analysis of a single MCI trial found that academic sites had lower rates of dropout compared to commercial sites [14]. Alternatively, a recent review of an antidepressant clinical trial found no significant difference in dropout between academic and non-academic sites [15]. Multinational trials are essential to regulatory goals and a thorough understanding of drug safety, but may carry increased risk of variability, including differences in the proportions of enrolled study partner dyad types by geographic region [16, 17]. Completion rates also may differ among global geographic regions in multinational trials [16].

The objective of this study was to quantify the relationship between study partner type and trial recruitment and retention in two multisite, industry-funded multinational registrational mild-to-moderate AD dementia trials and to assess whether previously found results in NIA-sponsored trials were replicated. We hypothesized that non-spousal dyads would be at higher risk for dropping out.

## Study methods

### Data source

**Fig. 1:**
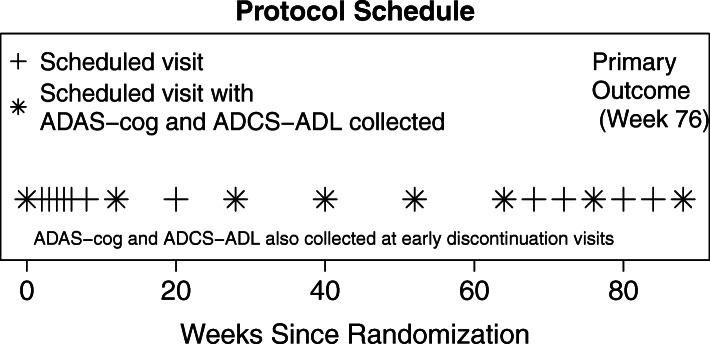
Protocol schedule. Alzheimer’s Disease Assessment Scale for cognition (ADAS-cog) and Alzheimer’s Disease Cooperative Study–Activities of Daily Living (ADCS-ADL) were scheduled to be collected at 0, 12, 28, 40, 52, 64, 76, and 88 weeks and at any early study discontinuation visits. The primary outcome was collected at week 76

We performed secondary data analyses of two multinational studies of semagacestat, an oral gamma secretase inhibitor, in mild-to-moderate AD dementia. The trials employed essentially identical inclusion criteria and protocols (ClinicalTrials.gov numbers: NCT00594568 and NCT00762411). Each trial enrolled subjects 55 years and older who met NINCDS-ADRDA diagnostic criteria for AD and had Mini-Mental State Examination (MMSE) scores between 16 and 26. The co-primary outcomes of the trials were the 76-week changes from baseline in Alzheimer’s Disease Assessment Scale cognitive subscale (ADAS-cog) and Alzheimer’s Disease Cooperative Study–Activities of Daily Living (ADCS-ADL) scale. ADAS-cog and ADCS-ADL scores were collected throughout the study and at visit 17 scheduled at week 76 (Fig. 1). Based upon a recommendation from the data and safety monitoring board (DSMB), the trials were stopped early for futility in August 2010 [18, 19]. Of 2648 randomized participants, 640 completed the primary endpoint. Among the remaining participants, 45 died, 908 were lost to follow-up, 2 screen failed, and 1053 were unable to complete due to sponsor decision.

The research protocols were approved by the Internal Review Board at each trial site, and written informed consent was collected from each participant or a surrogate. The current study did not access participant identifying information and therefore does not meet the criteria for human subjects research.

### Data analysis/statistical methods

Given the similarities between the protocols, we combined the two trials for analysis. We removed two subjects who reported a race of “other,” 41 subjects with missing covariate values, and two subjects who screen failed. Of the two subjects with a race of “other,” one was lost to follow-up and one completed the co-primary outcomes. Among the 41 subjects with missing covariates, 24 subjects were lost to follow-up, 12 had to withdraw from the study early due to the sponsor decision, and 5 completed the co-primary outcomes. Most of the missing data was confined to study partner characteristics and the Geriatric Depression Scale score. The final dataset used in this analysis included *N* = 2603 participants. We used the baseline Resource Utilization in Dementia (RUD) to classify participants based on the type of study partner with whom they enrolled. We categorized the nations in which participants enrolled into seven global regions, as done previously [16, 20]. The seven regions included North America, South America/Mexico, Eastern Europe/Russia, Western Europe/Israel, Australia/South Africa, Asia, and Japan. Descriptive statistics were stratified by study partner type and reported as mean (standard deviation) for quantitative variables and frequency (percent) for categorical variables unless otherwise stated.

We investigated the relationship between study partner type and retention by quantifying the time to early study exit using survival analysis. The outcome of our analysis was defined as the time from randomization to the first of (1) early study exit prior to completing the week 76 primary endpoint due to participant decision (*N* = 883) or (2) death (*N* = 45). Among the 45 subjects who discontinued due to death, we observed stretches of time of up to 4 months between final visit dates and death dates. Out of concern that subjects had decided to drop out prior to their death or that worsening health prevented them from attending visits, we classified these subjects as dropping out at their final in-person visit. Subjects with ADAS-cog and ADCS-ADL scores reported at visit 17 or on visit 16 or 18 and within weeks 74 to 78 were considered to have completed the trial and were censored at their week 76 visit. Participants who discontinued because of the sponsor’s decision to terminate the study based on the recommendation of the DSMB were censored at the time of study termination (*N* = 1041).

In the primary analysis, we used Cox’s proportional hazards regression model to estimate the relative risk (RR) for early study discontinuation associated with study partner type. We used a partial likelihood ratio test (LRT) to assess if the risk of early exit differed by study partner type. To account for potential confounding, we a priori decided to adjust for participant characteristics of sex, age, education, APOE4 carrier status, global region, baseline cognitive functioning (MMSE), potential vascular contributions to cognitive impairment (Hachinski Ischemic Score), and depression (Geriatric Depression Scale), as well as study partner sex, study partner age, and study partner employment status. We did not adjust for race and ethnicity or the proportion of time the study partner and participant spend together because of concerns about lack of model identifiability due to multi-collinearity with global region and study partner type, respectively. To assess the robustness of our results to the modeling decisions, we performed two sensitivity analyses. First, we considered early study exit due to death as a censoring event instead of an observed exit time. Second, we restricted our analysis to participants in North America and re-ran the primary analysis while adjusting for race and ethnicity.

In exploratory analyses, we investigated the extent to which individual covariates could explain the relationship between study partner type and dropout. We compared the unadjusted model, fully adjusted model, and models that only adjusted for one potential confounder at a time to determine if the study partner effect was primarily attenuated by a single confounding factor. Finally, we ran a random forest model for survival data with log-rank splitting to identify the top predictors of dropout with the highest variable importance values [21–23]. All analyses were performed in R version 3.6.0 [24].

## Results

Descriptive statistics of the trial participants are reported in Table 1. More than 65% of participants enrolled with a spouse as their study partner. Participants who enrolled with a spouse tended to be younger and were more likely to be male and non-Hispanic white when compared to those who enrolled with a non-spousal partner. Among global regions, similarly high proportions of spousal dyads were observed for North America, Western Europe/Israel, Australia/South Africa, Asia, and Japan. Higher proportions of non-spousal dyads were observed in Eastern Europe/Russia and South America/Mexico.

**Table 1:** Sample description reported for all randomized participants with a reported study partner type. Quantitative variables are summarized as mean (standard deviation) and categorical variables as *N* (%)

	Study partner type			Total^d^ (*N* = 2635)
	Spouse (*N* = 1729)	Adult child (*N* = 662)	Other (*N* = 244)	
Participant age	71.81 (7.9)	75.77 (7.8)	74.87 (8.4)	73.09 (8.1)
Education (years)	13.07 (3.9)	10.61 (4.1)	11.13 (4.1)	12.27 (4.1)
Sex: female	750 (43%)	519 (78%)	189 (77%)	1458 (55%)
**Region**^a^				
North America	722 (42%, 73%)	172 (26%, 17%)	89 (36%, 9%)	983 (37%)
South America/Mexico	87 (5%, 43%)	77 (12%, 38%)	39 (16%, 19%)	203 (8%)
Eastern Europe/Russia	111 (6%, 38%)	153 (23%, 53%)	25 (10%, 9%)	289 (11%)
Western Europe/Israel	413 (24%, 75%)	100 (15%, 18%)	36 (15%, 7%)	549 (21%)
Australia/South Africa	113 (7%, 74%)	22 (3%, 14%)	18 (7%, 12%)	153 (6%)
Asia	112 (6%, 55%)	74 (11%, 36%)	18 (7%, 9%)	204 (8%)
Japan	171 (10%, 67%)	64 (10%, 25%)	19 (8%, 7%)	254 (10%)
**Race/ethnicity**				
Caucasian	1364 (79%)	452 (68%)	174 (71%)	1990 (76%)
Asian	292 (17%)	142 (21%)	40 (16%)	474 (18%)
Hispanic	57 (3%)	59 (9%)	24 (10%)	140 (5%)
African American/Black	16 (1%)	9 (1%)	6 (2%)	31 (1%)
**Baseline assessments**				
Mini-Mental State Exam	20.94 (3.2)	20.06 (3.0)	20.66 (3.0)	20.69 (3.1)
ADAS-cog	23.06 (9.0)	24.7 (9.0)	23.16 (8.7)	23.48 (9.0)
Activities of Daily Living	61.08 (12.2)	57.42 (14.5)	58.72 (14.6)	59.95 (13.1)
Hachinski Score^b^	1 (0, 1)	1 (0, 1)	1 (0, 1)	1 (0, 1)
Geriatric Depression Scale^b^	2 (1, 3)	2 (1, 3)	2 (1, 3)	2 (1, 3)
**Study partner**				
Age	70.56 (8.7)	48.08 (9.0)	56.02 (14.5)	63.57 (13.7)
Contribution level^b,c^	5 (5, 5)	4 (3, 5)	4 (3, 5)	5 (4, 5)
Sex: female	972 (56%)	457 (69%)	200 (82%)	1629 (62%)
Paid job	385 (22%)	451 (68%)	119 (49%)	955 (36%)

^a^(Column %, Row %) are reported for region

^b^Summarized as median (25%, 75%)

^c^Contribution level among all caregivers is measured on a scale from 1 (1–20%) to 5 (81–100%)

^d^Two participants were excluded for having a race of 'other' and one was excluded for missing partner type

**Fig. 2:**
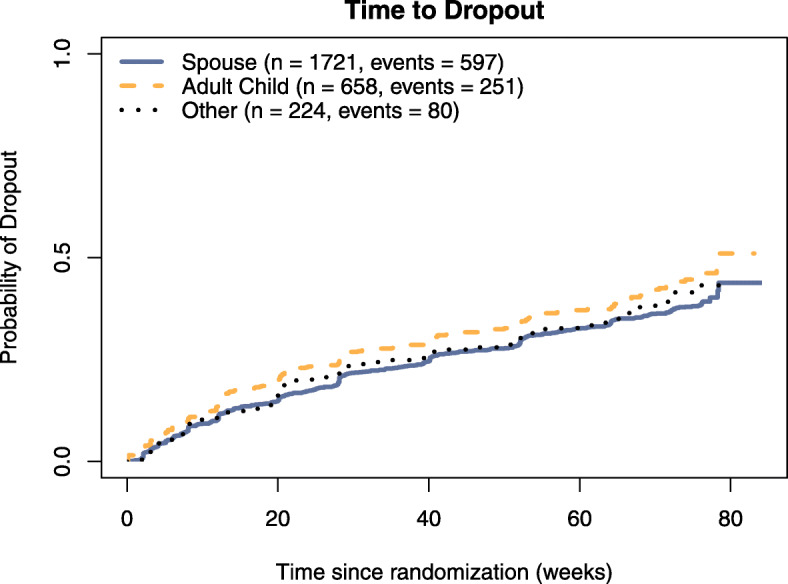
Kaplan-Meier survival curves for time to dropout. Probability of dropping out is stratified by study partner type (spouse, adult child, or other). Spousal study partners tend to dropout later

Among spousal dyads with complete covariates, 569 (33%) were lost to follow-up, 28 (2%) died, 655 (38%) were forced to halt participation due to sponsor decision, and 469 (27%) completed the week 76 co-primary endpoints. Among adult child dyads, 235 (36%) were lost to follow-up, 16 died (2%), 288 (44%) were forced to halt participation, and 119 (18%) completed the study. Among other dyads, 79 (35%) were lost to follow-up, 1 died (< 1%), 98 (44%) were forced to stop, and 46 (21%) completed. Dyad-specific Kaplan-Meier curves estimating the probability of early trial exit by time since randomization are presented in Fig. 2. In unadjusted models, we estimated that participants with an adult child study partner had a 23% (RR = 1.23; 95% CI 1.06, 1.42; *p* = 0.007) higher risk of dropping out compared to those with a spousal study partner; other dyads were estimated to have a 8% (RR = 1.08; 95% CI 0.86, 1.36; *p* = 0.524) higher risk of dropping out compared to spousal dyads. Although we observed a relationship between study partner type and early study exit in the unadjusted model, this relationship was no longer significant after adjusting for potential confounders. We estimated that subjects who enrolled with an adult child study partner had only a 6% (RR = 1.06; 95% CI 0.79, 1.43; *p* = 0.698) higher risk and other partners had a 3% (RR = 1.03; 95% CI 0.75, 1.40; *p* = 0.863) higher risk of dropping out compared to those who enrolled with a spousal study partner when adjusting for potential confounders (Table 2). Sensitivity analyses censoring subjects who died or limiting the analysis to participants in North America and adjusting for race did not substantially alter our results (Tables 3 and 4).

**Table 2:** Estimated relative risk (RR) for the risk of dropout

	Subjects (*N* = 2603)	Events (*N* = 928)	Deaths (*N* = 45)	Unadjusted RR	Unadj. *p* value	Adjusted RR	Adj. *p* value
**Partner type**^a^					0.027		0.928
Spouse	1721	597	28	1	–	1	–
Adult child	658	251	16	1.23 (1.06, 1.42)	0.007	1.06 (0.79, 1.43)	0.698
Other	224	80	1	1.08 (0.86, 1.36)	0.524	1.03 (0.75, 1.40)	0.863
Participant age (per 10 years)	2603	928	45	1.41 (1.29, 1.53)	< .001	1.41 (1.26, 1.58)	< .001
Education (years)	2603	928	45	0.98 (0.96, 1.00)	0.011	0.99 (0.97, 1.01)	0.165
Partner age (per 10 years)	2603	928	45	1.04 (0.99, 1.09)	0.15	1.02 (0.93, 1.13)	0.645
**Baseline test scores**							
Mini-Mental State Exam	2603	928	45	0.94 (0.92, 0.96)	< .001	0.95 (0.92, 0.97)	< .001
Hachinski Score	2603	928	45	1.14 (1.06, 1.22)	< .001	1.04 (0.96, 1.12)	0.336
Geriatric Depression Scale	2603	928	45	1.09 (1.05, 1.13)	< .001	1.09 (1.05, 1.14)	< .001
**Participant sex**							
Female	1437	493	19	1	–	1	–
Male	1166	435	26	1.07 (0.94, 1.22)	0.281	1.26 (1.05, 1.51)	0.013
**Partner sex**							
Female	1602	588	26	1	–	1	–
Male	1001	340	19	0.91 (0.80, 1.04)	0.172	1.10 (0.92, 1.32)	0.293
**Partner employed**							
No	1652	615	27	1	–	1	–
Yes	951	313	18	0.88 (0.77, 1.01)	0.06	0.93 (0.79, 1.09)	0.373
**APOE carrier**							
No	903	333	20	1	–	1	–
Yes	1700	595	25	0.94 (0.82, 1.08)	0.396	1.00 (0.87, 1.15)	0.993
**Region**							
North America	971	369	19	1	–	1	–
South America/Mexico	201	81	6	1.24 (0.98, 1.57)	0.08	1.03 (0.79, 1.34)	0.828
Eastern Europe/Russia	286	133	2	1.50 (1.24, 1.83)	< .001	1.44 (1.16, 1.80)	0.001
Western Europe/Israel	543	167	7	0.78 (0.65, 0.94)	0.007	0.82 (0.67, 0.99)	0.044
Australia/South Africa	151	57	6	0.96 (0.73, 1.28)	0.803	0.97 (0.73, 1.29)	0.823
Asia	200	62	2	1.07 (0.82, 1.40)	0.608	1.02 (0.77, 1.35)	0.899
Japan	251	59	3	0.60 (0.45, 0.79)	< .001	0.58 (0.44, 0.78)	< .001

^a^Partial likelihood ratio test of the null hypothesis that there is no difference in risk of dropping out by study partner (SP) type

**Table 3:** Sensitivity analysis—estimated relative risk (RR) for the risk of dropout when censoring participants who died

	Subjects (*N* = 2603)	Events (*N* = 928)	Deaths (*N* = 45)	Unadjusted RR	Unadj. *p* value	Adjusted RR	Adj. *p* value
**Partner type**^a^					0.57		0.954
Spouse	1721	569	28	1	–	1	–
Adult child	658	235	16	1.20 (1.03, 1.40)	0.018	1.03 (0.76, 1.39)	0.837
Other	224	79	1	1.12 (0.88, 1.41)	0.356	1.05 (0.77, 1.44)	0.756
Participant age (per 10 years)	2603	883	45	1.37 (1.26, 1.49)	< .001	1.37 (1.22, 1.54)	< .001
Education (years)	2603	883	45	0.98 (0.97, 1.00)	0.016	0.99 (0.97, 1.01)	0.205
Partner age (per 10 years)	2603	883	45	1.04 (0.98, 1.09)	0.188	1.02 (0.93, 1.13)	0.623
**Baseline test scores**							
Mini-Mental State Exam	2603	883	45	0.94 (0.92, 0.96)	< .001	0.95 (0.93, 0.97)	< .001
Hachinski Score	2603	883	45	1.12 (1.05, 1.21)	0.001	1.02 (0.95, 1.10)	0.586
Geriatric Depression Scale	2603	883	45	1.10 (1.05, 1.14)	< .001	1.10 (1.05, 1.15)	< .001
**Participant sex**							
Female	1437	474	19	1	–	1	–
Male	1166	409	26	1.05 (0.92, 1.20)	0.471	1.18 (0.98, 1.42)	0.085
**Partner sex**							
Female	1602	562	26	1	–	1	–
Male	1001	321	19	0.90 (0.78, 1.03)	0.135	1.04 (0.86, 1.25)	0.684
**Partner employed**							
No	1652	588	27	1	–	1	–
Yes	951	295	18	0.87 (0.75, 0.99)	0.042	0.91 (0.77, 1.07)	0.251
**APOE carrier**							
No	903	313	20	1	–	1	–
Yes	1700	570	25	0.96 (0.84, 1.10)	0.580	1.02 (0.88, 1.17)	0.821
**Region**							
North America	971	350	19	1	–	1	–
South America/Mexico	201	75	6	1.20 (0.94, 1.53)	0.146	1.00 (0.76, 1.31)	0.981
Eastern Europe/Russia	286	131	2	1.55 (1.27, 1.89)	< .001	1.49 (1.19, 1.87)	< .001
Western Europe/Israel	543	160	7	0.79 (0.65, 0.95)	0.012	0.82 (0.67, 1.00)	0.049
Australia/South Africa	151	51	6	0.91 (0.68, 1.23)	0.537	0.91 (0.67, 1.23)	0.525
Asia	200	60	2	1.08 (0.82, 1.42)	0.567	1.03 (0.77, 1.37)	0.854
Japan	251	56	3	0.60 (0.45, 0.79)	< .001	0.58 (0.43, 0.78)	< .001

^a^Partial likelihood ratio test of the null hypothesis that there is no difference in risk of dropping out by study partner (SP) type

**Table 4:** Sensitivity analysis—estimated relative risk (RR) for the risk of dropout for participants in North America

	Subjects (*N* = 2603)	Events (*N* = 928)	Deaths (*N* = 45)	Unadjusted RR	Unadj. *p* value	Adjusted RR	Adj. *p* value
**Partner type**^a^					0.595		0.53
Spouse	720	268	13	1	–	1	–
Adult child	171	69	6	1.13 (0.86, 1.48)	0.374	0.81 (0.49, 1.36)	0.427
Other	80	32	0	1.12 (0.78, 1.62)	0.532	1.06 (0.66, 1.72)	0.802
Participant age (per 10 years)	971	369	19	1.47 (1.30, 1.66)	< .001	1.47 (1.23, 1.75)	< .001
Education (years)	971	369	19	0.95 (0.92, 0.98)	0.001	0.97 (0.93, 1.00)	0.034
Partner age (per 10 years)	971	369	19	1.12 (1.02, 1.23)	0.021	0.97 (0.82, 1.14)	0.699
**Baseline test scores**							
Mini-Mental State Exam	971	369	19	0.94 (0.91, 0.97)	< .001	0.94 (0.91, 0.97)	< .001
Hachinski Score	971	369	19	1.12 (1.00, 1.26)	0.049	1.04 (0.92, 1.19)	0.513
Geriatric Depression Scale	971	369	19	1.08 (1.01, 1.15)	0.035	1.08 (1.01, 1.16)	0.031
**Participant sex**							
Female	504	188	8	1	–	1	–
Male	467	181	11	1.02 (0.83, 1.26)	0.821	1.02 (0.73, 1.42)	0.903
**Partner sex**							
Female	600	239	13	1	–	1	–
Male	371	130	6	0.86 (0.69, 1.06)	0.162	0.88 (0.64, 1.23)	0.469
**Partner employed**							
No	649	270	13	1	–	1	–
Yes	322	99	6	0.69 (0.54, 0.86)	0.001	0.75 (0.57, 0.99)	0.046
**APOE carrier**							
No	318	127	7	1	–	1	–
Yes	653	242	12	0.88 (0.71, 1.10)	0.263	0.96 (0.76, 1.22)	0.76
**Race/ethnicity**							
White	922	346	16	1	–	1	–
Asian	13	6	0	1.13 (0.54, 2.38)	0.741	1.15 (0.50, 2.63)	0.743
Hispanic	16	8	3	1.30 (0.65, 2.60)	0.452	1.07 (0.52, 2.19)	0.861
African American/Black	20	9	0	1.43 (0.70, 2.93)	0.325	1.18 (0.52, 2.68)	0.69

^a^Partial likelihood ratio test of the null hypothesis that there is no difference in risk of dropping out by study partner (SP) type

**Fig. 3:**
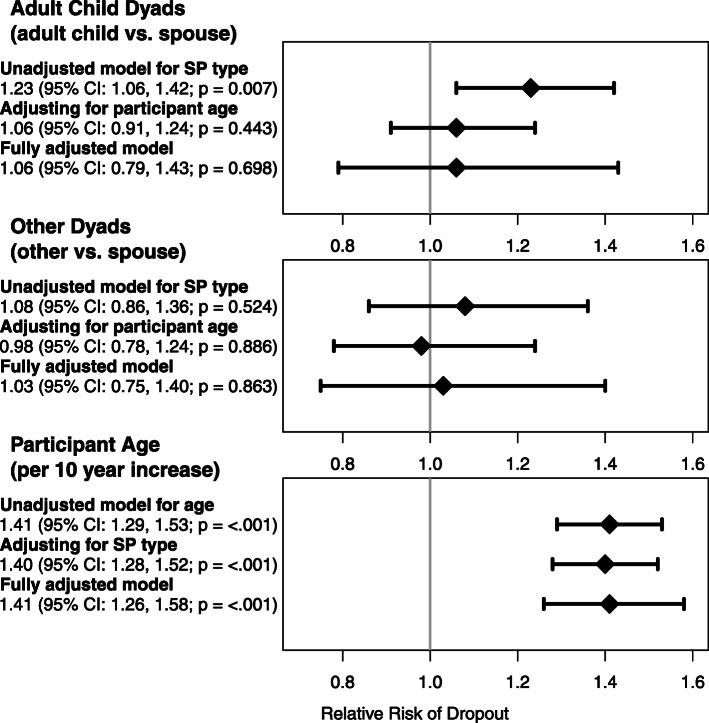
Forest plots of the estimated relative risk of dropping out comparing adult child dyads to spousal dyads (top panel) and other dyads to spousal dyads (middle panel). The bottom panel shows the estimated relative risk of dropping out comparing to subpopulations of participants differing in age by 10 years. Each of the three plots shows three estimates: an unadjusted model, a model adjusting only for study partner (SP) type or participant age, and a fully adjusted model containing all potential confounders. Notice that the relationship between study partner type and dropout is attenuated after adjusting for participant age, but the relationship between participant age and dropout remains after adjusting for study partner type and all potential confounders

When comparing the fully adjusted model, unadjusted model, and the 11 models with one adjustment variable, we found that adjusting solely for participant age almost completely attenuated the relationship between study partner type and the risk of early exit to the null (Fig. 3). In the models only adjusting for age, participants with adult child partners were estimated to have a 6% higher risk of trial incompletion (RR = 1.06; 95% CI 0.91, 1.24; *p* = 0.443) and other dyads were estimated to have a 2% lower risk of trial incompletion (RR = 0.98; 95% CI 0.78, 1.24; *p* = 0.886) when compared to participants with spousal partners. Age had a strong association with dropout even after adjusting for all other considered confounders (Fig. 3). Predictive analyses utilizing random forests estimated the variable importance for each covariate and ranged from 0.0285 to less than 0.0001. Participant age was the most predictive of trial incompletion with a variable importance of 0.0285, continent was the second most predictive with a variable importance of 0.0133, and the third most predictive variable was MMSE with a variable importance of 0.0048.

## Discussion

In two multinational industry-sponsored phase III trials of a candidate disease-modifying therapy for AD, we observed differences in the characteristics and behaviors of the study dyads when looking at unadjusted proportions. In several global regions, individuals with spousal study partners were overrepresented, compared to population estimates of caregivers. Participants with a spousal study partner were younger, more educated, more often male, and more often white than counterparts with non-spouse partners. They also dropped out of the trials less frequently than did participants with non-spousal partners. Overall, 35% of spousal dyad participants dropped out of the study or died, compared to 38% of participants with adult study partners and 36% of participants with other study partners. These results are consistent with previous observations in academic trials conducted exclusively in North America [10] and suggest that investigators may need to be alert to the increased risk of dropout for participants enrolling with a non-spouse partner.

Despite these observations in the raw data, multivariable analyses indicated that the increased risk of dropout among adult child dyads was not due to the study partner relationship, per se. Instead, the relationship between study partner type and dropout dissipated after controlling for participant age. This was true when the model outcome included or excluded participants lost due to death. Increased age may be a risk factor for dropout. In a previous study of AD trials, subjects were partitioned into age groups (55–65, 65–75, and > 75) and higher rates of dropout and dropout due to adverse events in the older two groups were observed [25]. Our results conflict with previous studies, which found that risk of dropout remained for non-spousal (other) dyads, even when controlling for age [10]. What factors may explain this discrepancy is unclear. The current trials were global, but restricting our analysis to participants from North America did not substantially alter results. The current trials were much larger, including a mix of academic and non-academic sites. These differences in trial design, size, and study population between the previous analysis and the current work may contribute to the differing conclusions. For example, previous analyses in similar large studies found that both participant marital status and site type were associated with participant retention [5, 14]. We lacked information here to examine site type or a potential interaction between site and dyad types. Alternatively, we cannot rule out that other unmeasured differences between the study populations, such as research attitudes (Stites et al., *in press*), could have contributed the differences in observations.

The lack of significant relationship between dyad type and dropout (when controlling for covariates) may be good news for AD trial investigators. Increasing enrollment of participants with non-spousal partners represents a potentially key opportunity to address the crisis in trial recruitment [3]. Moreover, doing so may allow for more representative and generalizable study samples, given that non-spousal caregivers are most common [7–10] and are particularly prevalent among racial and ethnic communities that are even more drastically underrepresented in clinical trials [6, 26].

AD trials now enroll a spectrum of patient populations, including those with mild cognitive impairment (“prodromal AD” if biomarker evidence of AD is present) and those with normal cognitive performance but biomarker evidence of disease (“preclinical AD”) [27]. Despite these diagnostic differences, all AD trials require dyadic participation and it will be important to examine whether similar relationships between study partner type and recruitment and retention are observed in preclinical and prodromal AD. To our knowledge, this matter is not well studied, though natural history studies suggest that dyad type may be similarly important in retention in predementia trials [28, 29].

To reduce missing data and subsequent bias, dropout must be minimized to the greatest extent possible. These results suggest that older participant age is a risk factor for this event. In previous meta-analytic studies, older participants in ADRD trials also demonstrated less decline on trial cognitive and functional outcome measures, potentially reducing trial power for demonstrating disease-slowing effects [30, 31]. Older participants may therefore bring added risks to trial integrity. Most AD patients, however, are over age 75 [7]. Recent NIH policies aim to ensure inclusion of older more representative samples in research, especially clinical trials [32]. Trialists may need to consider optimal means for retaining older participants to trial completion. In particular, efforts to reduce the burden of participation and incentivize trial completion should be pursued, including financial support throughout the trial and unique payment schedules if they improve completion rates [33, 34]. Such strategies may be particularly valuable for hard to recruit participants, notably including older participants, non-spouse dyads, and minority races and ethnicities [35]. Numerous other tactics for retaining participants exist [36], though few have been tested rigorously in the setting of trials, let alone for potential effect modification by participant (or study partner) characteristics.

## Limitations

This study had limitations. We are unable to establish a causal relationship between study dyad characteristics and dropout due to the observational nature of this study and the potential for unmeasured confounding factors. We, however, a priori hypothesized which variables would be potential confounders or were predictive of the response and accounted for any that were measured. Residual confounding may exist with respect to socioeconomic status, study partner education level, marital status, site type, study partner depression or caregiving burden, comorbidities, or attitudes toward research. Higher levels of closeness between caregivers and participants also may be related to study partner type and have been reported to be associated with slower functional and cognitive decline, fewer neuropsychiatric symptoms, lower care costs, and both better and worse health outcomes for the caregiver [37–40]. Racial and ethnic minorities were underrepresented in this study [10, 14], which may have led to bias in the estimated relationship between partner type and dropout. Lastly, a large number of participants were unable to complete participation in the trial due to the administrative decision of the sponsor to halt the study. These participants did not choose to drop out of the trial but did not complete the study. Since accrual patterns among these participants did not differ by dyad type (data not shown), this was unlikely to bias estimates.

## Conclusions

Previous analyses of smaller academic AD trials found that participants who enrolled with a spousal study partner were less likely to dropout. We did not fully replicate these findings within two multinational phase III studies. We instead found that age was both a strong predictor of who dropped out and a confounder of observed differences between dyad types that largely explained the relationship between study partner type and the risk of study incompletion. Future AD clinical trials need to prioritize including and retaining non-spousal dyads and older participants to prevent missingness and create more generalizable trial results.

## Data Availability

The data that support the findings of this study are available from Eli Lilly but restrictions apply to the availability of these data, which were used under license for the current study, and so are not publicly available. Data are however available from the authors upon reasonable request and with permission of Eli Lilly.

## Abbreviations

AD: Alzheimer’s disease
ADAS-cog: Alzheimer’s Disease Assessment Scale for cognition
ADCS-ADL: Alzheimer’s Disease Cooperative Study–Activities of Daily Living
APOE4: Apolipoprotein E ε4
CI: Confidence interval
DSMB: Data and safety monitoring board
LRT: Partial likelihood ratio test
MMSE: Mini-Mental State Examination
MCI: Mild cognitive impairment
NIA: National Institute of Aging
NINCDS-ADRDA: National Institute of Neurological and Communicative Disorders and Stroke and the Alzheimer’s Disease and Related Disorders Association
RR: Relative risk
RUD: Resource Utilization in Dementia
SP: Study partner
